# Transmission Models of Historical Ebola Outbreaks

**DOI:** 10.3201/eid2108.141613

**Published:** 2015-08

**Authors:** John M. Drake, Iurii Bakach, Matthew R. Just, Suzanne M. O’Regan, Manoj Gambhir, Isaac Chun-Hai Fung

**Affiliations:** University of Georgia, Athens, Georgia, USA (J.M. Drake, S.M. O’Regan);; Georgia Southern University, Statesboro, Georgia, USA (I. Bakach, M.R. Just, I.C.-H. Fung);; Monash University, Melbourne, Victoria, Australia (M. Gambhir)

**Keywords:** Ebola virus, viruses, modeling, transmission models, outbreaks

## Abstract

To guide the collection of data under emergent epidemic conditions, we reviewed compartmental models of historical Ebola outbreaks to determine their implications and limitations. We identified future modeling directions and propose that the minimal epidemiologic dataset for Ebola model construction comprises duration of incubation period and symptomatic period, distribution of secondary cases by infection setting, and compliance with intervention recommendations.

Mathematical models are used to generate epidemic projections under different scenarios, provide indicators of epidemic potential, and highlight essential needs for data. To aid the interventions in the 2014 Ebola epidemic in West Africa, in September 2014 we reviewed models of historical Ebola virus (EBOV) outbreaks (Table 1) and their estimated parameters (Table 2; Technical Appendix).

**Table 1:** Compartmental models of historical Ebola virus outbreaks

Feature	Model		
	Chowell et al. (*1*)	Lekone and Finkenstädt (*4*)	Legrand et al. (*5*)
Outbreak*	DRC 1995, Uganda 2000†	DRC 1995‡	DRC 1995, Uganda 2000§
Assumed			
Homogeneous random mixing	Yes	Yes	Yes
All human-to-human contact	Yes	Yes	Yes
Considered			
Nosocomial transmission	No	No	Yes
Burial transmission	No	No	Yes
No. transmission parameters	2 (preintervention decays to postintervention)	1 (decay to 0)	3 (community, nosocomial, burial)
Distribution	Exponential	Geometric	Exponential
Underreporting accounted for	No	No	No

*The DRC outbreak was caused by the Zaire strain; the Uganda outbreak was caused by the Sudan strain. DRC, Democratic Republic of Congo. †Data sources: DRC 1995 (*2*), Uganda 2000 (*3*). ‡Data source: DRC 1995 (*2*). §Data sources: DRC 1995 (*2*,*6–8*), Uganda 2000 (*3,9*).

**Table 2:** Estimated values of parameters as identified in the Ebola modeling articles*

Reference	Outbreak	Model	R_0_ estimate	Incubation period, d (SD)†	Infectious period, d (SD)
Chowell et al. (*1*)	DRC 1995	SEIR‡	1.83 (SD 0.06)	5.3 (0.23)	5.61 (0.19)
	Uganda 2000	SEIR‡	1.34 (SD 0.03)	3.35 (0.49)	3.5 (0.67)
Lekone and Finkenstädt (*4*)	DRC 1995	SEIR, MCMC (vague prior)	1.383 (SD 0.127)	9.431 (0.620)	5.712 (0.548)
	DRC 1995	SEIR, MCMC (informative prior)	1.359 (SD 0.128)	10.11 (0.713)	6.523 (0.564)
Legrand et al. (*5*)	DRC 1995	Stochastic compartmental model (SEIHFR)	2.7 (95% CI 1.9–2.8)		
	Uganda 2000	Stochastic compartmental model (SEIHFR)	2.7 (95% CI 2.5–4.1)		
Eichner et al. (*10*)	DRC 1995	Incubation period estimate based on parameterized lognormal distribution function		12.7 (4.31)	
Ferrari et al. (*11*)	DRC 1995	MLE	3.65 (95% CI 3.05–4.33)		
	DRC 1995	Regression	3.07§		
	Uganda 2000	MLE	1.79 (95% CI 1.52–2.30)		
	Uganda 2000	Regression	2.13§		
White and Pagano (*12*)	DRC 1995	MLE	1.93 (95% CI 1.74–2.78)		

*DRC, Democratic Republic of Congo; MCMC: Markov chain Monte Carlo; MLE, maximum-likelihood estimation; SEIR, susceptible-exposed-infectious-removed; SEIHFR, susceptible-exposed-infectious-hospitalized-funeral-removed. Blank cells indicate that no information was provided from the original study. †The incubation period for Ebola virus is believed to be the same as its latent period, i.e., infected persons become infectious only when symptomatic. ‡Combination differential equation model and Markov chain model. §Neither CIs nor SDs were provided in the study.

## The Review

Chowell et al. (*1*) developed a deterministic SEIR (susceptible-exposed-infectious-recovered) compartmental model and a stochastic continuous-time Markov chain version (Figure). A transmission coefficient, β_0_, was assumed to be constant before interventions and reduced transmission after intervention at a constant rate, β_1_. The model was fit to cases from the 1995 Democratic Republic of Congo (DRC) outbreak and the 2000 Uganda outbreak by using least squares. The final size was sensitive to the timing of control measures. The authors concluded that a 2-week delay in the timing of interventions would have increased the final size of the outbreak by a factor of 2.

**Figure:**
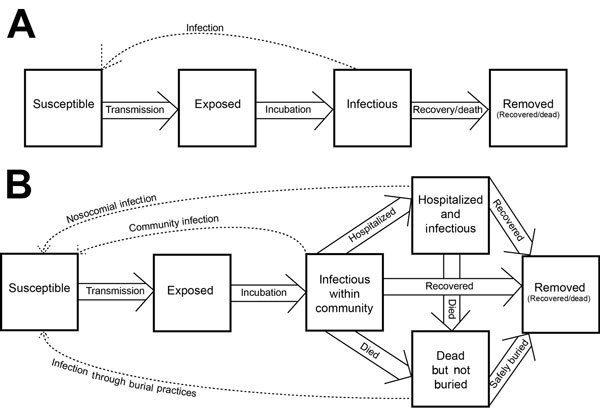
Conceptual diagrams illustrating Ebola SEIR and SEIHFR models of historical Ebola virus outbreaks. SEIR, susceptible-exposed-infectious-removed; SEIHFR, susceptible-exposed-infectious-hospitalized-funeral-removed.

Lekone and Finkenstädt (*4*) modified the model of Chowell et al. for discrete-time, stochastic progression. They fit their model to daily incidence and mortality time series from the 1995 DRC outbreak using Markov chain Monte Carlo. R_0_ was estimated by using vague and informative prior distributions. This exercise concluded that interventions shortened the epidemic from 950 days to 200 days and reduced total number of cases from 3.5 million to just over 300. Effective reproduction number (R_E_) was estimated to decrease to <1 five days after intervention onset.

Legrand et al. (*5*) accounted for transmission in different contexts through a stochastic model with 6 compartments: susceptible, exposed, infectious, hospitalized, dead-but-not-yet-buried, removed (Figure). Three transmission coefficients corresponded to community transmission, nosocomial transmission, and transmission at funerals. Interventions were assumed to be completely efficient from their onset: no transmission occurred at burials and hospitals, and community transmission was reduced by a multiplier estimated by model fitting. Parameters were estimated by fitting the model to incidence data (DRC, 1995; Uganda, 2000), by using approximate maximum likelihood, and an expression for R_0_ was derived. After interventions, community transmission was estimated to have been reduced to 88% and 12% of its initial value in the DRC and Uganda outbreaks, respectively, with respective R_E_ of 0.4 (95% CI 0.3–0.6) and 0.3 (95% CI 0.2–0.4). The authors acknowledged that the 95% CIs around transmission and efficacy estimates were wide and conducted a sensitivity analysis of intervention parameters. This analysis indicated that community transmission was key to epidemic dynamics in Uganda, whereas funerals contributed more to transmission in the DRC. Rapid hospitalization significantly reduced community transmission and barrier nursing practices along with effective isolation of Ebola patients controlled the epidemics (*5*).

These models (*1*,*4*,*5*) shared certain features. They assumed homogeneous mixing of the population, exponentially or geometrically distributed incubation and infectious periods, and a sudden decay in transmission after intervention. None accounted for underreporting. Future exercises should explore the consequences of these assumptions. With ideal data, fitted models would be stress-tested to assess their validity, for instance challenging models to predict out-of-fit data.

Three additional studies estimated incubation period or R_0_ by using statistical models. Eichner et al. (*10*) assumed a log–normally distributed incubation period. Ferrari et al. (*11*) used maximum likelihood with a chain binomial distribution and regression to estimate R_0_. White and Pagano (*12*) assumed that the number of secondary cases produced by a patient followed a Poisson distribution, with expected value R_0_.

Collectively, these studies underscore that practical decisions in modeling dictate trade-offs between fitting to limited data and explicit representation of reality, including interventions. A model with a single transmission rate might fit well to data but might not be useful for decision making that evaluates intervention effects in different transmission contexts. A model with 3 transmission rates might represent transmission in community, nosocomial, and funeral contexts (e.g., [*5*]), but the 3 parameters are unlikely to be uniquely identifiable. Because EBOV typically amplifies during nosocomial transmission, a model with 2 transmission parameters (community transmission, comprising funeral and household transmission in 1 parameter, and nosocomial transmission) might represent the best compromise. Such a model would enable interventions, such as personal protective equipment and efficient hospitalization of persons with community-acquired EBOV infection, to be considered.

Other features are important for understanding the probable paths of small outbreaks. These include nonexponential incubation and infectious periods (*13*) and individual heterogeneity in the generation of secondary infections, including “super-spreaders” (*14*). The models reviewed are approximations to these processes. The extent to which these approximations introduce bias could be understood by developing a range of models, perhaps using versions of the chain binomial model or other generalized contagion processes.

Another issue that has not been studied is the role of spatial scale. All extensive EBOV outbreaks involved multiple scales of transmission. At the smallest scale, persons most at risk for infection are those caring for an Ebola patient. Understanding these household contacts helps estimate outbreak size. Human settlements constitute a “household of households.” Transmission occurs among households in communities, at hospitals, or at funerals. Understanding these between-household contacts is needed to determine the outbreak’s extent. Finally, understanding connections between settlements by human movements is needed to determine the paths and speed of large-scale spatial spread and therefore the total infected area and domain for surveillance and monitoring. Although the assumption of population homogeneity can be justified for models of historical EBOV outbreaks, given the limited geographic extent of those outbreaks, models for the 2014 outbreak might need to address heterogeneity in population density and human movements because of the extensive geography involved.

Two issues new to the 2014 EBOV epidemic are underreporting and compliance. To assess underreporting, perhaps comprehensive contact tracing can be performed in a small number of locales and extrapolated. If cases can be identified through 2 independent routes, then case matching can be used to identify the total number of cases (*15*). Concerning compliance, the fraction of patients admitted to hospitals and, of those remaining in the community, the fraction of decedents with safe burials should be identified. Compliance of personal protective equipment among health care workers is central to understanding the role of nosocomial transmission.

## Conclusions

Model fitting is craft as well as science. Modeling demands decisions, including what mathematical representations to use, the type and magnitude of variation to be considered, and the values that can be taken as given versus the values still to be estimated. In the face of data scarcity, we suggest that construction of models of the 2014 outbreak would have benefited from a minimal dataset that included 1) the mean and variance of the incubation period and symptomatic period, respectively; 2) the probability distribution of secondary cases by infection setting; and 3) compliance with recommendations. For secondary cases, in addition to the average, the commonness of outliers (super-spreaders), the frequency of zeros, and the variance in the distribution need to be known.

Technical Appendix Methods for study of the transmission models of historical Ebola outbreaks.
