# Supplementary material for: Transmission Models of Historical Ebola Outbreaks
Source: Emerg Infect Dis. 2015 Aug;21(8):1447–50. doi: 10.3201/eid2108.141613 (PMC4517740; doi:10.3201/eid2108.141613)
Supplement: Technical Appendix — Methods for study of the transmission models of historical Ebola outbreaks. [file 14-1613-Techapp-s1.pdf]

Article DOI: http://dx.doi.org/10.3201/eid2108.141613

Transmission Models of Historical Ebola Outbreaks

Technical Appendix

Methods

We limited our scope to compartmental models of Ebola transmission in human populations of historical outbreaks before 2014. Models of global transmission, transmission from and between wildlife animals, within-household transmission, and agent-based models were excluded from this review because they are not directly relevant to our focus.

Data for this review were identified by searches through PubMed by using EndNote X4 software's PubMed search interface and references from relevant articles. Only English language papers were reviewed. Search items (in title or abstract) included: "Ebola" and 4 different variations of the word "model": "model," "models," "modeling," and "modelling." Initially 470 titles were identified but after removing duplicates that the number diminished to 337. After screening their titles and abstracts, we selected 14 papers for full-text retrieval. Papers that were not relevant to mathematical modeling of Ebola (such as "animal models") were excluded. The references of the selected papers were screened again to identify possible relevant papers. Another 37 papers were identified by their titles. After a second round of screening, 11 of the 51 papers were retrieved for full text reading; 40 were deemed irrelevant and were excluded. After reading their full text, only 3 dynamic modeling papers and 3 statistical modeling papers were included for detailed review (Technical Appendix Figure).

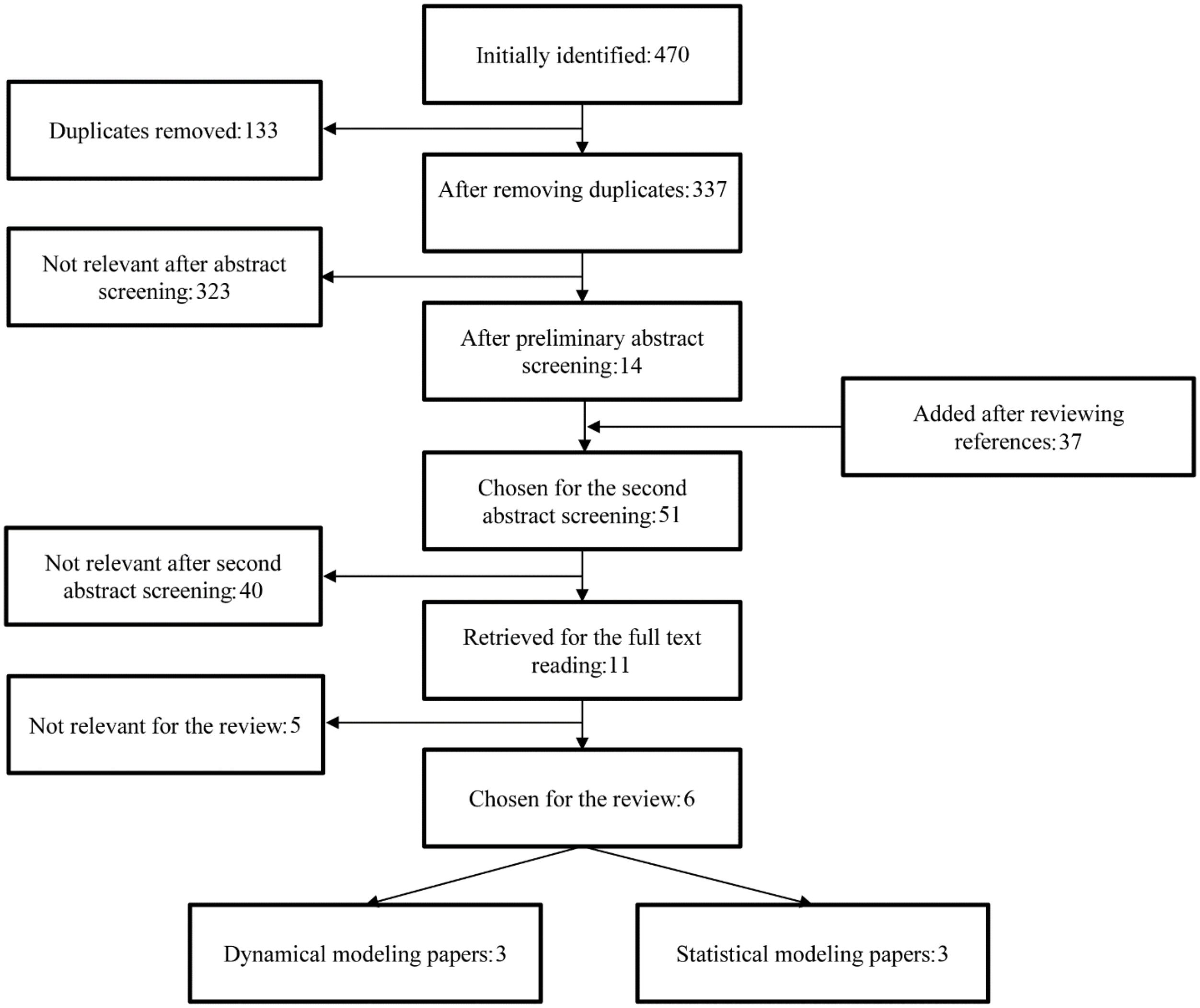

Technical Appendix Figure. Flow diagram of the literature screening process.
